# Annotations

**Published:** 1909-10-02

**Authors:** 


### Annotations.

### The Late Dr. Hubert Biss.

We regret to announce the death, at the age of 37, of Dr. Hubert Elwyn Jones Biss, M.A., M.D., D.P.H., who for some time past had acted as the London representative upon the editorial staff of our contemporary the *Medical Press and Circular*. Dr. Biss died at Eastbourne on September 22, after a painful illness, and his early death is a loss to the medical profession, to medical journalism and to a wide circle of friends in London and elsewhere. He had considerable personal charm and much energy and ability, and all who knew him must regret deeply the cutting short of so promising a career. The late Mr. Gladstone, during his last illness, was attended professionally by Dr. Hubert Biss, who for six months was in residence at Hawarden Castle for this purpose. Dr. Biss studied medicine at Cambridge University, University College, London, and the Middlesex Hospital, and qualified in 1896, taking that year the English Conjoint Diploma and the M.B., B.C. degrees of his University. Subsequently he obtained his M.A. and M.D. Cantab degrees and the diploma of Public Health of Edinburgh and Glasgow. Dr. Biss took much practical interest in the medical aspects of life insurance, and was medical referee and examiner to several large assurance companies. He was also at the time of his death Lecturer on Ambulance to the L.C.C. His past appointments included demonstratorships of anatomy and biology at Cambridge, and resident posts at the Brompton Hospital for Consumption; while for five years he acted as assistant medical officer at the fever hospitals of the Metropolitan Asylums Board of London. Dr. Biss had throughout a strong literary taste and power of expression. He contributed many papers on a variety of subjects to British professional journals, and published several small books and pamphlets, besides a great deal of unsigned journalistic work for the periodical of which he was assistant editor.

### Medical Bills in Parliament.

Sir William Collins is to be heartily congratulated upon the success that has attended the Metropolitan Ambulances Bill, which was read a third time and passed on Tuesday night last. The Bill now merely awaits the royal assent to become law. The supporters of the Bill have worked hard, and the success that has crowned their efforts has not been achieved without combating some opposition, while Sir William himself has shown considerable tact and statesmanship in the management of his Bill, which should win him the practical recognition and support of his constituents. An equally excellent measure accompanied the Ambulances Bill to the higher chamber, where it met with an equally favourable reception and also passed a third reading. We refer to the Cinematograph Bill, on the merits of which we have already commented in a previous issue. Both these Bills are very necessary ones, and they will appeal to everyone who is interested in the welfare of the community. It is interesting to note that Sir William Collins stands sponsor to the Asylums Superannuation Bill, which provides for the granting of superannuation allowances to officers and servants employed in asylums for the insane in Great Britain and Ireland. The Bill, which was presented to the House of Commons in August last, has been somewhat delayed by press of more urgent Parliamentary business, but it comes up for second reading before the Lords next week, and it is sincerely to be hoped that its passage will be as smooth and as successful as that of the medical Bills already referred to.

### The Soldier's Teeth.

Considerable public and parliamentary interest appears to have been aroused by a recent newspaper report to the effect that a batch of soldiers with defective teeth has been drafted out of the Army because the War Office refused to go to a somewhat considerable expense in refitting the men with teeth. In the House of Commons several questions on the subject have been put to the Secretary of State for War. Mr. Ward wished means to be devised whereby the defective teeth of recruits, rejected because of such dental defects, could be rectified. Mr. Lynch, in whom the medical profession should have an enthusiastic parliamentary supporter, desired that provision to be made for the proper care of soldiers' teeth as a matter of daily routine. Mr. Remnant wanted to know if the newspaper report already alluded to was true, and Mr. Lupton, who finds the trail of the serpent of compulsory vaccination over every evil or regrettable incident that is mooted in Parliament, volunteered the statement that many dentists considered the prevalence of dental caries as a result of his pet abomination. Mr. Haldane in his replies was vague and could throw little light on the question. Whenever, in the opinion of a recruiting medical officer, dental treatment will render a recruit efficient, the necessary treatment is now given, provided the cost of such treatment does not exceed £1. That was the present position, and the War Office could really not provide false teeth for 50,000 men, nor apparently could it concern itself actively with the proper care of already enlisted men as a routine duty. We may point out that the matter is not so trifling as the Secretary of State for War appears to hold it, and that only by such proper and routine attention as Mr. Lynch suggested will his department be saved the necessity of providing false teeth for regiments of men at a cost of £3 per man or of dismissing otherwise physically fit soldiers because they happen to suffer from toothache.

